# The role of glycans in the mechanobiology of cancer

**DOI:** 10.1016/j.jbc.2023.102935

**Published:** 2023-01-21

**Authors:** Anurag Purushothaman, Mohammad Mohajeri, Tanmay P. Lele

**Affiliations:** 1 Department of Biomedical Engineering, Texas A&M University, Houston, Texas, USA; 2 Department of Biomedical Engineering, Texas A&M University, College Station, Texas, USA; 3 Artie McFerrin Department of Chemical Engineering, Texas A&M University, College Station, Texas, USA; 4 Department of Translational Medical Sciences, Texas A&M University, Houston, Texas, USA

**Keywords:** Glycans, Mechanobiology, Proteoglycans, Cancer, Collagen, AGE, advanced glycation end product, CREB, cyclic adenosine monophosphate response element-binding protein, ECM, extracellular matrix, FAK, focal adhesion kinase, GAG, glycosaminoglycan, HA, hyaluronan, HSPG, heparan sulfate proteoglycan, RAGE, receptor for advanced glycation end products, RUNX1, runt-related transcription factor 1, SLRPs, small leucine-rich proteoglycans, TAZ, transcriptional coactivator with PDZ-binding motif, TME, tumor microenvironment, UDP, uridine diphosphate, YAP, yes-associated protein

## Abstract

Although cancer is a genetic disease, physical changes such as stiffening of the extracellular matrix also commonly occur in cancer. Cancer cells sense and respond to extracellular matrix stiffening through the process of mechanotransduction. Cancer cell mechanotransduction can enhance cancer-promoting cell behaviors such as survival signaling, proliferation, and migration. Glycans, carbohydrate-based polymers, have recently emerged as important mediators and/or modulators of cancer cell mechanotransduction. Stiffer tumors are characterized by increased glycan content on cancer cells and their associated extracellular matrix. Here we review the role of cancer-associated glycans in coupled mechanical and biochemical alterations during cancer progression. We discuss the recent evidence on how increased expression of different glycans, in the form of glycoproteins and proteoglycans, contributes to both mechanical changes in tumors and corresponding cancer cell responses. We conclude with a summary of emerging tools that can be used to modify glycans for future studies in cancer mechanobiology.

A tumor is a growing population of aberrant somatic cells. Over the course of tumorigenesis, the properties of the tumor microenvironment (TME) change dramatically. The progression of many solid cancers involves the recruitment of several different cell types to the TME, such as endothelial cells that form “leaky” blood vessels (1), cancer-associated fibroblasts, and mesenchymal stromal cells that secrete extracellular matrix (ECM) proteins such as collagen and glycans such as hyaluronan (2, 3, 4), and immune cells that secrete different cytokines, proteases, and reactive oxygen species (5, 6, 7). These changes alter not only the biochemical composition of the TME but also the mechanical stiffness of the ECM. Increased collagen secretion results in the assembly of higher concentration of collagen fibrils, which results in a mechanically stiff ECM. In addition, nonenzymatic glycation of collagen and resulting accumulation of advanced glycation end products (AGEs) can increase stiffness of collagen independently of its concentration, by increasing the cross-links between collagen fibrils (8). Such mechanical changes are common across different types of solid tumors and are responsible for the presentation of solid tumors as stiff lumps within the tissue (9). Stiffening of the ECM, whether due to increased matrix deposition or due to increased matrix cross-linking, can enhance cancer cell growth, disrupt tissue structure and polarity, and promote cell migration (8, 10, 11, 12, 13, 14, 15, 16, 17, 18, 19, 20).

Cells can sense mechanical cues from their microenvironment, a process termed mechanosensing, and adapt to these cues through changes in signaling pathways and gene expression, a process termed mechanotransduction (21). The simplest and robust canonical response of cells to ECM stiffening is increased cell spreading. Cell spreading is mediated by transmembrane integrin receptors, which preferentially cluster on stiff ECM compared with soft ECM, to form focal adhesions. Integrin clustering and focal adhesion formation is reciprocally coupled with the activation of signaling pathways including the Rho/Rho kinase (ROCK) pathway, PI3 kinase signaling, and YAP signaling (10, 22, 23, 24, 25) that in turn impact cell migration, traction force generation, and cell proliferation.

Glycans have recently emerged as important mediators and/or modulators of mechanotransduction. The term “glycan” refers to carbohydrate-based polymers that either exist as free molecules such as hyaluronan or covalently bound to lipids or proteins. Protein-bound glycans fall into two classes, glycoproteins and proteoglycans (26, 27). Glycoproteins are proteins that carry one or more branched glycan chains bound to either asparagine residues (*via* nitrogen linkages to form *N*-linked glycans) or serine/threonine residues (*via* oxygen linkages to form *O*-linked glycans) of the polypeptide (Fig. 1). The *N*- and *O*-linked glycans on glycoproteins can become further modified by terminal glycan motifs containing fucose (Lewis antigens) or sialic acids (28) catalyzed by fucosyltransferases (29) and sialyltransferases (30), respectively. Proteoglycans consist of a core protein covalently bound to one or more glycan chains called glycosaminoglycans (such as chondroitin sulfate or heparan sulfate chains). Glycolipids consist of glycan chains bound to a lipid ceramide (glycosphingolipid), where the first sugar bound to the ceramide can be either glucose (glucosylceramide) or galactose (galactosylceramide) (Fig. 1). Most of the glycoproteins/proteoglycans or glycolipids are either bound to the cell membrane or shed/secreted by the cells to become a part of the extracellular matrix (31).

**Figure 1:**
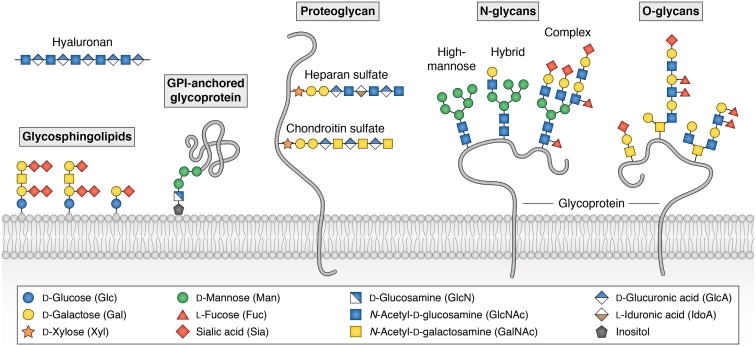
**Schematic representation of different glycans involved in cancer progression**. Glycans either exist as free forms or are attached to various proteins or lipids on the cell surface. Glycosphingolipids consist of the lipid ceramide linked to a variable series of sugars that can be further modified with terminal sialic acids. Glycoproteins carry one or more branched glycan chains attached to either asparagine residues (*via* nitrogen linkages to form *N*-linked glycans) or serine/threonine residues (*via* oxygen linkages to form *O*-linked glycans) of the polypeptide. *N*-glycans share a common pentasaccharide core that is extended by high mannose, or both mannose and GlcNAc residues (hybrid) or complex types containing branched structures containing two or more antennae. *O*-Glycans are initiated by GalNAc with elongated GlcNAc containing glycans. Both *N*- and *O*-glycans are further modified by terminal sialic acid residues. Some glycoproteins called the glycosylphosphatidylinositol (GPI)-anchored proteins are anchored in the outer leaflet of the plasma membrane by a glycan linked to phosphatidylinositol. Glycosaminoglycans are linear polysaccharides consisting of repeating disaccharide units that exist as either free sugar chains (such as hyaluronan) or mostly found attached to proteoglycans (such as heparan sulfate or chondroitin sulfate chains). Sugars are represented by colored geometric symbols.

Changes in glycan structure and content in tumors are a hallmark of cancer progression (32). This review focuses on the role of glycans in mediating cellular sensing of and response to changes in the mechanical stiffness of the tumor ECM, as well as in modulating the mechanical properties of the tumor ECM.

## Mechanobiological pathways in cancer

Mechanotransduction pathways are complex, involving a range of molecular players and mechanisms of action. Cells adhere to the ECM through transmembrane integrin receptors whose extracellular domains bind to the ECM (33) and whose intracellular domains associate with cytosolic proteins like talin, resulting in the assembly of a focal adhesion complex (21). Proteins in the adhesion like talin, zyxin, vinculin, and alpha-actinin can bind to the F-actin network (34). The focal adhesion can thus transmit forces generated in the cytoplasm by myosin-dependent contraction of F-actin filaments (35, 36, 37), through integrins, to the ECM, resulting in a traction stress on the ECM (38, 39, 40). The traction allows cells to maintain adhesion to the ECM as well as to move. Importantly, these integrin-mediated sites of adhesion are also key protein hubs that transmit externally applied mechanical force across the cell membrane (41) and are sites of transduction of the mechanical force into biochemical signaling, which ultimately impacts gene expression (21).

For a cell that is adherent to the ECM, a mechanical change, such as a mechanical stretch of the ECM, causes transmission of force through the integrin receptors, through talin and other linker proteins, to the cytoskeleton. The force can unfold talin and other proteins in the adhesion (42, 43), exposing cryptic binding sites for F-actin and structural proteins like vinculin, resulting in a higher local concentration of proteins localized to the adhesion including enzymes like focal adhesion kinase (FAK) and increased numbers of bonds with the actomyosin cytoskeleton. This remodeling response results in stronger adhesion, which is a classical adaptive response of cells to external mechanical force. The adaptive response, which can be triggered cell-wide (44), can in turn cause higher traction on the ECM, and remodel the ECM itself, through force-dependent unfolding of ECM proteins (45). Force applied to integrin receptors activates the small GTPase Rho signaling pathway, further stimulating actomyosin force generation, by activating its effector mDia1 (a mammalian homologue of *Drosophila* Diaphanous protein), which promotes actin filament polymerization (46, 47). Local force application to integrins can activate other enzymes like Src kinase and their downstream signaling (48). Forces applied to integrins can also modulate signaling pathways through the activation of different ion channels in the plasma membrane (49).

The ECM-integrin-cytoskeleton linkage is key to sensing and transduction of changes in the mechanical stiffness of the ECM into intracellular responses (50, 51). Cell migration, proliferation, differentiation, and tissue structure are profoundly altered on soft ECM compared with stiff ECM (10, 51). Adhesions and traction forces are small on soft ECM compared with stiff ECM, resulting in lower cell spreading on soft substrates (50, 52). The decrease in cell spreading on soft ECM correlates with rounded nuclear morphologies on soft ECM (53) and a lack of translocation of Yes-associated protein (YAP)/transcriptional coactivator with PDZ-binding motif (TAZ), transcriptional regulators of the hippo signaling pathway, to the nucleus (54, 55). YAP/TAZ get phosphorylated upon activation of the hippo pathway and are retained in the cytoplasm, while inactivation of the hippo dephosphorylates YAP/TAZ and causes their nuclear translocation where they induce gene expression (56). Interestingly, the sensitivity of YAP/TAZ translocation to ECM stiffness is independent of the hippo pathway. In fact, diverse mechanical stimuli impact YAP/TAZ translocation to the nucleus (57, 58), highlighting its importance as a mechanotransduction pathway.

## Altered glycan expression and cancer progression

The concentrations as well as the compositions of the different types of glycans are significantly altered in cancer (Fig. 1) and are specific to the type of cancer. Altered glycan composition and concentration impacts cancer cell adhesion, signaling, migration/invasion, tumor angiogenesis, drug resistance, epithelial to mesenchymal transition, and cancer metastasis (see reviews on this topic (59, 60, 61, 62, 63, 64)). Changes in glycation can serve as diagnostic biomarkers for some cancers (65, 66). Important proteoglycans that are upregulated in cancer include syndecans (67, 68), glypicans (69), perlecans (70, 71, 72), agrins (61, 73, 74, 75, 76, 77), small leucine-rich proteoglycans (SLRPs) such as biglycans (78, 79), lumicans (80, 81, 82, 83), and decorin (84). In addition, high levels of the glycosaminoglycan (GAG) hyaluronan in the ECM are common in many cancers (85, 86). Abnormally high *N*- and *O*-linked glycosylation of glycoproteins contribute to the development and progression of cancer (60). Increased fucosylation or sialyation of glycoproteins and increased glycation of lipids can both promote cancer progression (87, 88).

The increased glycation of proteins and lipids in cancer is broadly due to increased expression of specific glycosyltransferases, which are enzymes responsible for the initiation and elongation of glycan chains. An additional mechanism that may contribute to increased glycation is synthesis of UDP-GlcNAc by the nutrient-sensing hexosamine biosynthetic pathway (89). This pathway is known to be overactivated in cancer cells compared with normal cells (90), and the end product UDP-GlcNAc is the key substrate that is used for synthesis of glycans, including O-GlcNAcylation, which is a single sugar conjugation (91). O-GlcNAcylation is a dynamic and reversible posttranslational glycosylation that involves the addition of an N-acetylglucosamine (GlcNAc) from the precursor UDP-GlcNAc to the serine or threonine residues of a variety of cytoplasmic or nuclear proteins. A wide range of processes such as enzyme activity, protein stability, activation of survival signals, and chemoresistance in cancer are regulated by O-GlcNAcylation (92).

## Impact of glycans on mechanobiological pathways in cancer

Recent studies have shown that glycation of proteins can modulate pathways that mediate cellular response to mechanical cues from the ECM (Table 1) (59, 61). Below we discuss the role of different classes of glycans in modulating tumor cell response to mechanical changes in the ECM. We focus on the role of proteoglycans because most studies of the role of glycans in cancer mechanobiology have been performed in the context of proteoglycans.

**Table 1:** Glycans regulated mechanical properties and cancer types

Glycan type	Mechanical properties	References	Cancer type
Sialylation	Increases integrin tension, enhances maturation of focal adhesions and spreading and migration of cancer cells	(139)	Ovarian
Mucins	Promote integrin clustering, activate focal adhesion kinases	(135, 137)	Breast, glioma
Hyaluronan	Promotes ECM stiffening, supports accumulation of mechanical forces in tumor tissues	(166, 171, 172, 211, 212)	Glioma, breast, prostate
Agrin	Promotes ECM stiffening, activates the YAP/TAZ pathway, stabilizes focal adhesion complexes	(74, 95, 96)	Liver
Syndecans	Cytoplasmic domain binds to PDZ domain containing proteins such as syntenin-1 and recruits signaling and cytoskeletal proteins to the plasma membrane, promotes cytoskeletal rearrangements, cell–ECM interactions, migration, and metastasis	(213, 214, 215, 216, 217, 218)	Breast, lymphoma, myeloma, pancreatic, lung, glioma, ovarian
Syndecan-4	Enhances syntenin-1 binding, coordinates focal adhesion dynamics, promotes formation of stress fibers and mechanosignaling	(107, 108, 110) (219, 220)	Breast, glioma osteosarcoma, liver, kidney
Syndecan-1	Promotes formation of signaling complexes in cooperation with αVβ3, αVβ5, and α4β1 integrins, promotes ECM assembly and cancer cell spreading and migration, mediates flow stress–induced changes in cell shape	(112, 113, 114, 115, 149, 221, 222, 223, 224)	Myeloma, breast
Glypican	Mediates flow shear stress–induced nitric oxide synthase activation	(221)	Vascular endothelium
Decorin	Collagen fibril formation	(225)	
Biglycan	Promotes the formation of a denser collagen architecture, increases tissue stiffness, upregulates β1 integrin expression, promotes cancer cell invasion	(142)	Melanoma
Aggrecan	Prevents ECM stiffening in cartilage and aortic tissues	(226, 227)	
Serglycin	Promotes FAK and YAP signaling	(128)	Breast
AGEs	Promotes collagen stiffening	(8, 19, 24, 147, 148, 228)	Prostate, breast

ECM, extracellular matrix; FAK, focal adhesion kinase; YAP, yes-associated protein.

## Proteoglycans

### Agrin

Agrin is a heparan sulfate proteoglycan (HSPG), expressed either as a cell membrane proteoglycan or secreted into the ECM (93, 94). Agrin, which is expressed by several tissues, is known to cluster acetylcholine receptors in the neuromuscular junctions. It binds to lipoprotein-related receptor-4 (Lrp4) and mediates muscle-specific kinase (Musk) signaling in neuromuscular junctions. A few studies have implicated a role for agrin in cancer. Agrin expression is elevated in oral squamous cell carcinoma and promotes tumor aggressiveness (75). Agrin is overexpressed and secreted by hepatocellular carcinoma cells (95), activating the Lrp4/Musk signaling pathway and promoting the assembly of cell-ECM adhesions (95). Knockdown of agrin impairs adhesion assembly, similar to the effect of myosin inhibition (95). This suggests that agrin may promote actomyosin force-dependent assembly of cell-substrate adhesions, which is a crucial component of the cellular response mechanisms to mechanical cues from the ECM (36, 51). Indeed, agrin levels are higher in cells cultured on stiff 2D collagen-coated polyacrylamide hydrogels than in cells cultured on soft 2D polyacrylamide hydrogels (96). Knockdown of agrin prevents the nuclear translocation of YAP on stiff ECM, while addition of recombinant agrin causes the translocation of YAP to the nucleus even on soft ECM (96). Overall, the HSPG agrin is required for cancer cell response to mechanical stiffness of the ECM in hepatocellular carcinoma cells.

### Syndecans

Syndecans are a four-member family of transmembrane proteoglycans that predominantly carry heparan sulfate GAG chains. Almost all cells, except for erythrocytes, express at least one member of the syndecan family (97). A marked alteration of syndecan expression occurs in cancer with syndecans acting either as a tumor suppressor or promoter depending upon the cancer types. Loss of syndecan-1 expression in most epithelial tumors such as cervical, lung, head and neck, squamous cell, and esophageal cancers is associated with tumor progression and reduced patient survival (98, 99, 100, 101), suggestive of a tumor suppressive role for syndecan-1. In contrast, increased syndecan-1 expression in breast, pancreatic, ovarian, thyroid, and endometrial tumors is associated with tumor progression and poor prognosis (102). Importantly, both the core protein and the heparan sulfate chains of cell-surface or shed syndecans contribute to cancer progression (103, 104, 105).

There is evidence that syndecan-4, a member of the four-member family of transmembrane HSPGs, can act as a transmitter of mechanical force in fibroblasts and pancreatic stellate cells (106, 107). When pulsatile forces were applied to magnetic beads coated with a fragment of fibronectin that binds heparan sulfate chains on syndecan-4, or to beads coated with antibodies toward the core protein of syndecan-4, there was a reduction in bead displacement upon sustained force application, suggesting a mechanical stiffening response. This is similar to the stiffening response upon force application to integrins. Application of force to syndecan-4 altered the conformation of its cytoplasmic domain, promoting the binding of α-actinin, a scaffold protein that localizes to cell-ECM adhesions and also binds to F-actin (107). This linkage is likely responsible for the local mechanical stiffening response. Importantly, force application to syndecan-4 triggered the diffusion of phosphatidylinositol-3,4,5-trisphosphate (PIP3) lipid second messengers, which in turn activated β1 integrins cell-wide by binding to kindlin-2 and promoting RhoA mediated actomyosin contractility (107). The promotion of adhesion assembly by mechanical activation of syndecan-4 is consistent with another report that syndecan-4 is required for assembly of focal adhesions and stress fibers in fibroblasts (108, 109) and that syndecan-4 null mouse fibroblasts have reduced focal adhesions and matrix contraction abilities (110). A similar requirement for syndecan-4 has been reported in melanoma cells for contractility-dependent mechanosignaling (111). In addition, the extracellular core protein domains of syndecans 1 and 4 can bind to different integrin receptors—αvβ5, αvβ3, α3β1, and α4β1—forming diverse combinations (112, 113, 114, 115, 116) that could facilitate mechanosensitive formation of focal adhesion complexes and downstream activation of signaling pathways. Thus, the syndecan-4 mediated mechanotransduction pathway is likely to be broadly important in cancer, as the expression of syndecan-4 is high in cancers such as glioblastoma (117) and osteosarcoma (118), and the high expression correlates with reduced survival.

### Serglycin

Serglycin is the only known intracellular proteoglycan, with a core protein rich in serine–glycine repeats (119). The GAG chain of serglycin can be either heparin or chondroitin sulfate depending on the cell type in which serglycin is expressed. Although serglycin is considered as an intracellular proteoglycan, recent studies have shown that serglycin can be secreted by cancer cells and bind to cell surface receptors (120, 121, 122). Initial studies reported increased levels of serglycin only in hematological malignancies like multiple myeloma and leukemia; however, more recent findings suggest that serglycin is overexpressed in glioma and tumors of the breast, prostate, lung, and liver (123, 124, 125, 126, 127). Although the extent to which serglycin contributes to mechanotransduction remains understudied, a recent study reported that upregulation of serglycin expression in chemoresistant breast cancer cells activates FAK signaling (128), indicating a potential connection between serglycin and mechanotransduction (129). Serglycin upregulates YAP expression in breast cancer cells by activating integrin α5/FAK/CREB signaling. YAP in turn upregulates histone deacetylase 2 (HDAC2) expression *via* the transcription factor runt-related transcription factor 1 (RUNX1) to maintain stemness and chemoresistance in these cells (128). In addition, YAP positively regulates serglycin expression to form a feed-forward circuit in breast cancer cells. These findings highlight that the proteoglycan serglycin can mediate cancer cell adaptation to the changing mechanical environment through the FAK/YAP signaling axis; a possibility that deserves further exploration.

## Glycoproteins

### Mucins

Mucins are a family of highly glycosylated transmembrane glycoproteins produced by various epithelial cells and are categorized into membrane-associated mucins, gel-forming mucins, and soluble mucins. The striking feature of cell surface mucins is their long, densely glycosylated ectodomain that can extend hundreds of nanometers from the plasma membrane. Mucins form a gel-like mucus on the surface of the cells and impact integrin clustering, force sensing, and signaling (130). Mucins contribute to the bulk of the cancer-associated glycans (131, 132), and high amount of *O*-glycosylation on the central region of mucins makes them resistant to degradation during cancer progression. Cell surface MUC1 (mucin 1) and MUC16 (mucin 16) are consistently upregulated in epithelial cancers and are considered as biomarkers of the disease (133, 134). By virtue of their length, mucins can sterically modulate force on matrix-ligated integrin receptors, thereby activating them (135). Trimming mucins in glioblastoma cells, for example, represses integrin signaling, while increasing their size promotes tension-dependent integrin signaling (136). The dense glycan chains of mucins also promote cancer cell metastasis by enhancing integrin-FAK mechanosignaling and cell cycle progression by the PI3K-AKT axis (135, 137).

### Sialylation

While glycans clearly impact mechanotransduction, the function of particular sugar modifications like sialylation of proteins remains understudied. Sialylation is a form of glycosylation that involves the addition of sialic acid to the terminal end of *N*- and *O*-linked glycan chains by sialyltransferase enzymes. Aberrant sialylation is a driver of malignant phenotype and regulates cell–cell and cell–matrix interactions, proliferation, invasion, angiogenesis, resistance to apoptosis, and immune suppression (138). A recent study reported that sialylation of epidermal growth factor receptor enhances tension on ECM-ligated integrins (139). Using DNA-based tension probes and high-resolution total internal reflection fluorescence microscopy, the study showed that high sialyltransferase activity increases forces on the ECM and promotes maturation of focal adhesions, and eventual spreading and migration of ovarian cancer cells (139). These outcomes were driven by membrane retention and activity of epidermal growth factor receptor *via* sialylation and downstream activation of the ERK and PI3K-AKT signaling cascades. As sialylation is increased in many cancers (138), these findings highlight the need for further exploration of specific sugar modifications on proteins in the context of cancer cell mechanotransduction.

## Glycans and tumor ECM structure and mechanics

There is a general overexpression of proteoglycans on tumor cells and in the ECM in cancer (140). Proteoglycans in the ECM can bind to a wide range of matrix proteins such as collagen, fibronectin, and laminin, resulting in a more cross-linked matrix, and hence a stiffer matrix. For example, in breast cancer, even before tumor development there is an increased deposition of collagen I and proteoglycans in the ECM leading to increased breast density, which is a risk factor for breast cancer (141). Increased collagen and/or proteoglycan deposition results in matrix stiffening (141, 142), and this in turn creates a tumor supporting matrix that contributes to the pathology of the tumor.

Accumulation of AGEs in tissues has recently gained attention because of their significant role in inflammation and tumor development (143, 144). AGEs are formed when the carbonyl groups of endogenous reducing sugars (such as glucose-6-phosphate or ribose) nonenzymatically react with the free amino groups of proteins (145, 146). AGE-induced cross-links can stiffen the collagen matrix with minimal changes to the fiber architecture (19, 147). AGE-mediated ECM stiffening promotes epithelial cell invasion and decreases prostate cancer survival (148). Stiffening of collagen gels by glycation, but not by increased density, enhances the angiogenic outgrowth and branching of endothelial cell spheroids (8), suggesting a potential role for collagen glycation in tumor angiogenesis. Furthermore, a recent study reported that glucose can also promote glycation of collagen, AGE accumulation, and stiffening of collagen without any change in collagen fiber density or architecture (20). Expectedly, glucose-induced collagen stiffening promotes cancer cell contractility elongation and migration. The use of an AGE breaker alagebrium chloride (ALT711) reduces AGE-mediated matrix stiffening and cancer cell migration, without altering collagen pore size, chemical composition, or architecture (20). These findings demonstrate that AGE formation may be one mechanism by which diabetes promotes cancer and disruptions of AGEs using AGE-breaker drugs can be therapeutically beneficial.

In addition, HSPGs such as syndecan-1 can promote the assembly of parallel fibronectin and collagen-1 fibers facilitating the directional migration of cancer cells (149). The heparin II–binding domain on fibronectin binds with heparan sulfate chains of various members of the HSPG family (150). The binding of the heparan sulfate chains of syndecan-1 to the heparin-II domain may facilitate the unfolding of dimeric fibronectin and expose fibronectin self-assembly sites promoting fibrillogenesis. Furthermore, SLRPs biglycan and decorin can also modulate collagen fibril structure, fiber realignment, and matrix assembly (151, 152). Biglycan expression in melanoma promotes the formation of a denser collagen architecture leading to increased tissue stiffness (142). Biglycan-induced tissue stiffness in turn upregulates β1 integrin expression and promotes the invasion of melanoma cells (142). Biglycan can bind with different collagen subtypes, and a deficiency of biglycan can lead to collagen fibers becoming more loosely organized (153, 154, 155, 156, 157). Decorin is necessary for collagen fibril formation/realignment and in maintaining tissue stiffness (151, 158, 159), although its role in cancer development is less clear.

The GAG hyaluronan (HA) is a nonsulfated GAG chain with no core protein and therefore is not considered a proteoglycan. HA is overexpressed in most cancers, and accumulation of high levels of HA in the ECM triggers cancer progression and is associated with poor clinical outcome (85, 160, 161, 162, 163, 164, 165). Elevated ECM stiffness in patients with glioma is associated with a substantial increase in the levels of HA and the HA-binding glycoprotein tenascin C, but not collagen (166). The increased ECM stiffness is associated with poor prognosis in patients with glioma (166). HA accumulation can contribute to growth-induced solid mechanical stress and increase interstitial pressure through a retention of water within the tumor tissue due to the high negative charge on HA (167, 168, 169, 170, 171). Interestingly, soft HA substrates by themselves can elicit phenotypes from glioma cells like those when glioma cells are cultured on stiff ECM protein-coated substrates (172), suggesting that HA may trigger mechanotransduction pathways independently of ECM stiffness. Hyaluronan can thus contribute in diverse ways to the mechanical changes in tumors.

In addition to their role in sensing extracellular mechanical cues and translating them to biochemical changes, cell surface glycans can induce membrane curvature and hence control plasma membrane architecture (173). Glycosaminoglycans and glycoproteins can generate crowding pressure strong enough to induce plasma membrane curvature, bending membranes into different shapes. This may contribute to the formation of functional cell surface structures such as microvilli, filopodia, and lamellipodia, which are important for invasive migration, drug resistance, signaling, and secretion of extracellular vesicles (173, 174, 175, 176). More studies are needed to understand the extent to which glycan-induced membrane changes/instability contribute to cancer cell functions like invasive migration.

## Tools for targeting glycans

Our understanding of the function of glycans in cancer progression, and particularly, in mechanobiology of cancer is steadily growing, but many more studies are needed if we are to ultimately develop effective therapeutic strategies directed against glycan-mediated cancer mechanoadaptation. Effective tools to inhibit glycosylation are still lacking with the major challenge being limited specificity. Here we discuss the variety of tools that have emerged to alter glycan chains for cell biology studies. Toward the end we discuss glycan-targeting drugs used in clinical trials.

### Tools to target glycans for *in vitro* applications

The small molecules that inhibit glycosylation prevent the formation of glycosylation precursors, inhibit the activity of glycan processing enzymes, or act as primers and decoys (177, 178, 179). Small synthetic glycan mimetics can compete for glycan-binding sites and inhibit their binding and signaling. A glutamine analogue, 6-diazo-5-oxo-L-norleucine, is an example of a small molecule inhibitor used to inhibit the formation of glucosamine. All of the major glycan families require *N*-acetylglucosamine or *N*-acetylgalactosamine for their synthesis, and inhibiting glucosamine synthesis alters glycan assembly. For example, tunicamycin inhibits glycosylation of glycoproteins entirely by inhibiting *N*-glycosylation. Tunicamycin blocks the transfer of GlcNAc-1-phosphoate to dolichol phosphate, by inhibiting GlcNAc phosphotransferase, during the first steps of *N*-glycan synthesis (180). Importantly, this drug induces apoptosis in cancer cells by blocking *N*-glycosylation in various cell surface glycoproteins.

Many sugar analogues have been used to block *N*- and *O*-glycosylation (179). The basic premise is that these sugar analogues can inhibit glycosyltransferase enzymes by serving as donor and substrate analogues. Sugar analogues that have been developed include analogues of sialic acid such as fluorinated sialic acid mimetic (181), analogues of GalNAc such as per-acetylated 4F-GalNAc (182), analogues of GlcNAc such as 4F-GlcNAc (183), and analogues of fucose such as 2-fluorofucose, 5-alkynylfucose (184), and xylose (185). Xylosides mimic the xylosylated serine residues on the core protein on PGs, where the priming of GAG chains occurs. Xylosides therefore divert the assembly of GAG chains from the core protein of PGs and thereby inhibit PG formation (186, 187). Strategies to terminate the elongation of glycan chains have also been employed. For example, compounds such as mannosamine prevent the elongation of glycan chains on the GPI anchor and thereby block the incorporation of GPI glycans to GPI-anchored proteins (188). Likewise, oligomers of the GAG hyaluronan inhibit growth of several types of tumors by displacing endogenous hyaluronan from its receptor (189).

Rapid and stable knockout of individual or multiple glycogenes using genome editing tools is another effective method to prune the glycan chains (190). This method can be used to target a specific glycan by silencing specific glycotransferases or the entire glycan by silencing the chain initiating enzyme. For example, silencing specific enzymes such as sialyltransferase or fucosyltransferase inhibits the formation of sialofucosylated glycans on leukocytes and thereby blocks it binding to selectins (191, 192). In addition to genome editing, tool kits for membrane incorporation of fully synthetic polymers that mimic key features of glycoproteins have been developed for precision editing of sugar chains (193). The approach is to engineer the structure and composition of the cellular glycans using genetically encoded glycoproteins and expression systems. This toolkit has been used to manipulate the shape and functions of the glycans (194). Likewise, a tool kit based on CRISPR-Cas9 has been developed to prune specific glycan types, such as *N*-linked and *O*-linked glycans of glycoproteins and glycolipids (195). This gene editing tool kit may be a powerful approach for targeting specific glycans that regulate tumor mechanics.

Receptor for advanced glycation end products (RAGE) are multiligand-specific receptors that bind AGEs and are upregulated in cancer (196). Numerous RAGE inhibitors have been identified to target the AGE/RAGE axis in cancer (197, 198, 199, 200).

### Glycan-targeting drugs that progressed to clinical evaluation

Many highly sulfated and structurally defined heparan sulfate GAG analogues, which in part mimic the natural ligand, have been developed and tested in cancer (201, 202, 203). Heparin mimetics have high potential as anticancer agents due to their ability to inhibit heparanase activity, by competing with the heparan sulfate GAGs for binding/signaling growth factors and chemokines. The heparin mimetic, PI-88, is in clinical trials for melanoma and liver cancer (204). Utilizing the technology underpinning PI-88, a set of heparan sulfate mimetics, called the PG500 series, such as PG545, have been developed to target both angiogenesis and heparanase activity (205). Viable approaches to target the GAG chains include the use of enzymes that can cleave specific GAGs. The use of a humanized mutant chondroitinase ABC enzyme (ChaseM) that depolymerizes chondroitin sulfate chains on a variety of chondroitin sulfate proteoglycans enhances glioma cell sensitivity to chemotherapeutic drugs (206). The recombinant hyaluronidase (rHuPH20) can cleave the polymeric HA into substituent units (207). Depletion of HA by a PEGylated form of rHuPH20, PEGPH20, induced antitumor response and enhanced efficacy of anticancer drugs in preclinical models (208, 209, 210).

While a number of tools have now been developed as described above, tools are needed to specifically alter glycans. Such tools could greatly enhance understanding and targeting of glycans in cancer mechanobiology.

## Conclusions

**Figure 2:**
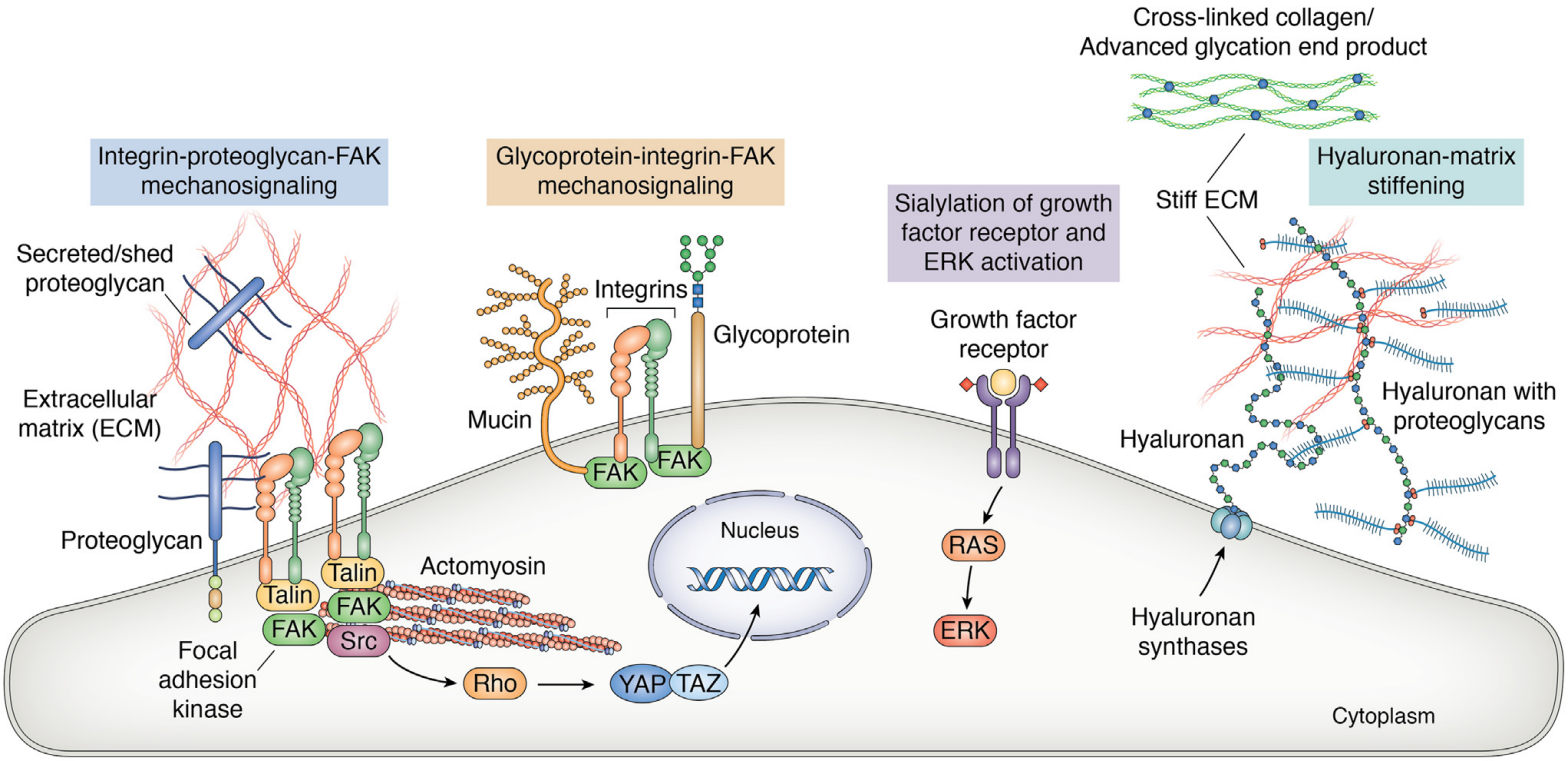
**Schematic illustration of mechanobiological pathways**. Mechanotransduction in cancer cells is mediated by complex molecular pathways involving glycans, integrins, focal adhesion proteins, and the actomyosin cytoskeleton. Cancer cells engage extracellular matrix (ECM) proteins through the heterodimeric integrin receptors, enabling the assembly of cell–ECM adhesions. Integrin receptors function as mechanotransmitters, transmitting force through adhesion proteins to the actomyosin cytoskeleton. Glycans can act as mechanotransmitters themselves or modify mechanotransduction pathways including integrin clustering, adhesion formation, cytoskeletal remodeling, and YAP/TAZ translocation to the nucleus. Crowding of glycans can also induce morphological changes in the plasma membrane. Furthermore, binding of glycans to matrix proteins can promote matrix realignment and assembly, which in turn can promote the stiffening of matrix. Accumulation of glycans such as hyaluronan in the ECM can increase interstitial pressure through retention of water within the confined tumor and thereby can contribute to mechanical stress. In addition, accumulation of advanced glycation end products can induce cross-linking and stiffening of collagen with minimal changes to the collagen fiber architecture. FAK, focal adhesion kinase; ECM, extracellular matrix.

Stiffening of the ECM is common in cancer. Cancer cells sense and transduce mechanical stiffness of the ECM into intracellular responses by a process called mechanotransduction, which promotes aberrant cell functions and contributes to cancer progression. There is mounting evidence that a wide range of glycans, including proteoglycans like syndecans, agrin, serglycin, SLRPs and others, hyaluronan, sialylated proteins, and *O*-linked glycans, are involved in cancer cell mechanotransduction. Glycans modulate multiple steps in the mechanotransduction pathway, including integrin ligand binding and clustering, adhesion assembly, cytoskeletal dynamics, and activation of the Rho/ROCK and YAP/TAZ pathway (Fig. 2) and correlated cancer cell functions such as migration, proliferation, and drug resistance. Some glycans can directly stiffen the ECM by forming more cross-links between ECM fibrils. We conclude that stiffening of tumor, as a normal part of cancer progression, is likely due directly to specific changes in glycan content/composition of the tumors. More comprehensive investigations of glycans in cancer cell mechanotransduction as well as development of new and more specific tools to modulate glycans both *in vivo* and *in vitro* will provide avenues for improved or novel cancer diagnostics and treatments in the future.

## Conflict of interest

The authors declare that they have no conflicts of interest with the contents of this article.
